# Radioresistant Extramedullary Plasmacytoma of the Maxillary Sinus: A Case Report and review article

**Published:** 2015-07

**Authors:** Matin Ghazizadeh, Hesamodin Alavi Amlashi, Golfam Mehrparvar

**Affiliations:** 1 *Department of Otorhinolaryngology, Head and Neck Surgery, Taleghani Hospital,Shahid Beheshti University of Medical Sciences, Tehran, Iran.*

**Keywords:** Plasmacytoma, Paranasal sinus neoplasms, Radiotherapy, Multiple myeloma

## Abstract

**Introduction:**

Plasmacytoma is a monoclonal proliferation of plasma cells. It can be an isolated lesion, for which the term extramedullary plasmacytoma is used, or a representation of multiple myeloma.The upper respiratory tract is the most common site for an extramedullary plasmacytoma. Sinonasal plasmacytomas cause different symptoms depending on the sites of origins and the areas of involvement. The treatment of choice for extramedullary plasmacytoma is local radiotherapy. Although it is generally accepted that plasmacytomas are radiosensitive, there are reports of cases that do not respond to radiotherapy.

**Case Report:**

A case of a 24-year-old male diagnosed with radioresistant extramedullary plasmacytoma of the maxillary sinus, who responded to surgical treatment, is reported.

**Conclusion:**

It is reasonable to consider an interdisciplinary approach in the management of extramedullary plasmacytoma. Considering early surgical intervention in cases encompassing risk factors of radiotherapy resistance is especially recommended before debilitating complications emerge.

## Introduction

Plasma cell myeloma and related plasma cell neoplasms are derived from a post germinal center B cell that has undergone somatic hypermutation (1). Plasmacytoma is a monoclonal proliferation of plasma cells. It can be an isolated lesion, for which the term extramedullary plasmacytoma is used, or a representation of multiple myeloma. Solitary plasmacytoma is characterized by a mass of neoplastic monoclonal plasma cells either in the bone (Solitary plasmacytoma of bone: SBP) or in the soft tissue (solitary extramedullary plasmacytoma: SEP) and without evidence of systemic disease that can be attributed to myeloma (2). 

Solitary extramedullary plasmacytoma accounts for less than 2% of all neoplastic plasma cell dyscrasias and can occur in any part of the body especially in the head and neck. These tumors usually remain localized and progression to myeloma is uncommon (3,4).

The upper respiratory tract is the most common site for an extramedullary plasmacytoma (80% of cases). These tumors are more common in the nose and nasopharynx, although any upper airway site can be involved (5). 

Solitary plasmacytoma of the maxilla is a rare condition that focuses solely on myelomatous tissues and is not disseminated to other parts of the skeleton (6).

A case of huge extramedullary plasmacytoma of the maxillary sinus with continued growth despite radiotherapy, which was resected completely by a combined approach, is reported.

## Case Report

About one year ago, a 24-year-old male was referred to the clinic by an oncologist. He had a two-year history of slow progressive right hemifacial swelling accompanied by right nasal obstruction. He also complained of epiphora of the right eye, right side rhinorrhea, and hemifacial pain. There was no history of hearing loss, aural fullness, visual impairment or diplopia, and the patient reported no constitutional symptoms.

The physical examination revealed generalized swelling of the right cheek region, which was firm and mildly tender upon palpation. There was adhesion of the lateral cheek skin to underlying tissues. The right nasolabial fold was obliterated. Ophthalmologic exam showed mild proptosis of the right eye and the right palpebral fissure was narrowed. However visual acuity was normal and funduscopic evaluation was unremarkable. Pupils were normal size and reactive to light. Extraocular movements were full in both eyes.

**Fig1:**
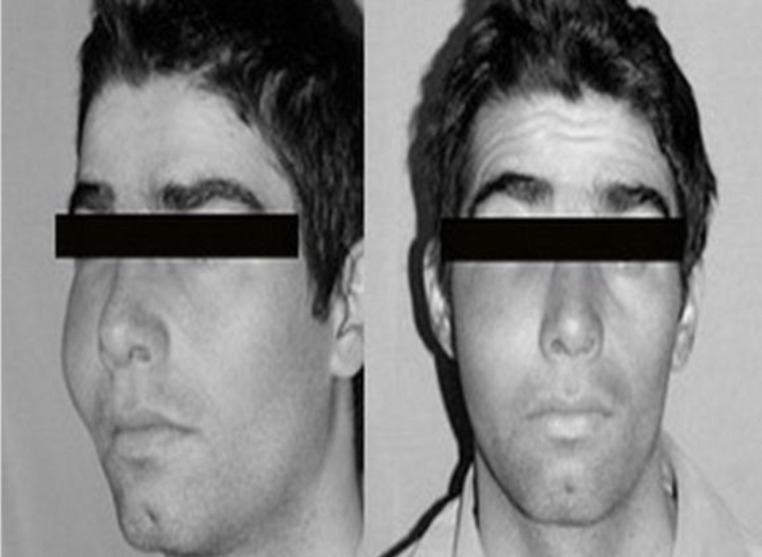
Patient Photograph showing right maxillary swelling

**Fig 2:**
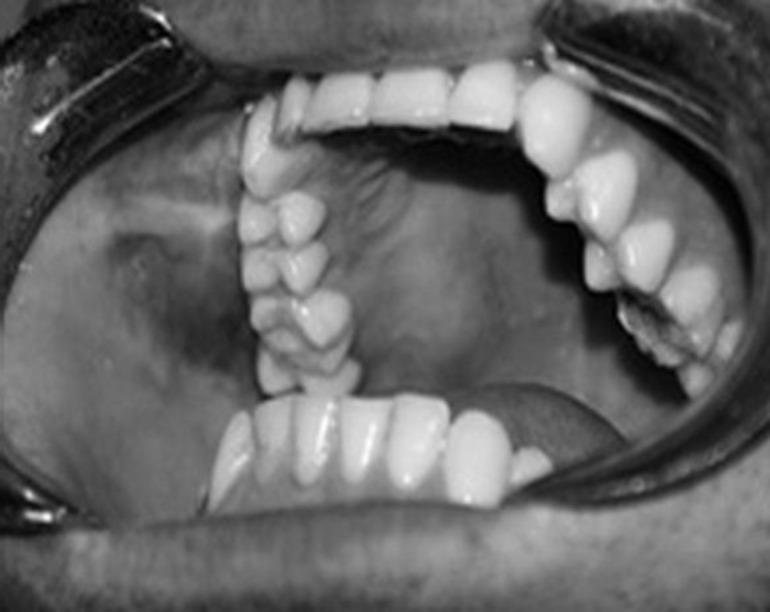
Intraoral examination revealed bulging of right hard palate and buccal alveolar sulcus

Intraoral examination showed bulging of the right hard palate and a bucal alveolar sulcus; but the mucosa remained intact and there were no loose teeth (Figs 1,2).

Rhinoscopy showed the medialized lateral nasal wall with deviation of the nasal septum to the left side and decreased size of left nasal cavity.

Neck examination revealed no regional lymphadenopathy and the remainder of the ENT examination was unremarkable. 

**Fig 3:**
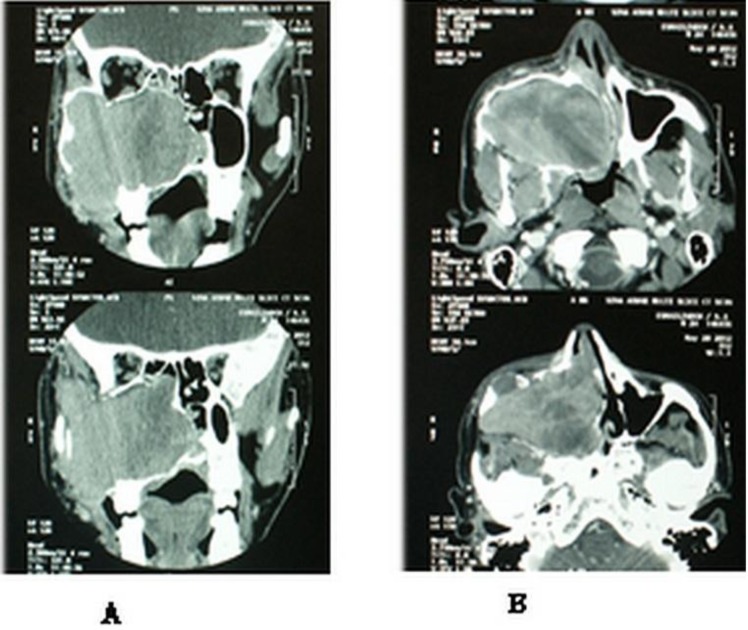
PNS scan coronal (A) and axial (B) view with contrast showing a soft tissue mass with heterogeneous enhancement, expanding maxillary sinus walls pushing the nasal septum to the left side

PNS CT scan showed a large well-defined expansile soft tissue mass in the right maxillary sinus with heterogeneous enhancement. It also showed destruction in the medial and lateral walls with remodeling and some erosions of the anterior and inferior walls in addition to thinning of the orbital floor and posterior wall (Fig.3).

The patient had undergone endonasal biopsy of the mass 1.5 years ago and histological examination had shown a small round blue cell tumor, positive for CD138, and compatible with plasmacytoma.

Full blood count, urea, creatinine, liver function tests, calcium, phosphorus, and electrolytes were within normal limits. Serum protein electrophoresis was normal and Bence Jones proteins were not present in the urinanalysis.

Bone marrow examination showed normal cellularity with 3% plasma cells. The serum B2 microglobuline level was normal.

Thoracolumbar spine MRI did not show any lesions in the bone or soft tissue.

The patient had received 28 sessions of radiotherapy but no improvement had been seen and the mass had continued to grow despite radiotherapy.He underwent surgical resection of the tumor. After the onset of general anesthesia, local infiltration with 1% xylocaine and 1 in 100000 adrenaline was performed. A Weber-Ferguson incision was made and the skin flap was elevated. There were erosions and extreme thinning of the anterior wall along with some subcutaneous tumoral extensions. The medial and lateral walls were destroyed by the tumor. Partial maxillectomy was performed while preserving the palate, infraorbital rim, and orbital floor. 

Extension of the tumor into adjacent lateral orbital soft tissue was resected; and at the end of the procedure, this region, as well as the ethmoid cavity, was assessed endoscopically (using straight and angled endoscopes) to look for any possible remnants of the tumor. The maxillary antrum was packed with antibiotic soaked ribbon gauze. The tumor was a well-defined, soft, friable, dark-red, 8*10 cm mass, which was sent for pathological examination. The diagnosis of extramedullary plasmacytoma was confirmed after pathological and immunohistochemical evaluation. 

**Fig 4:**
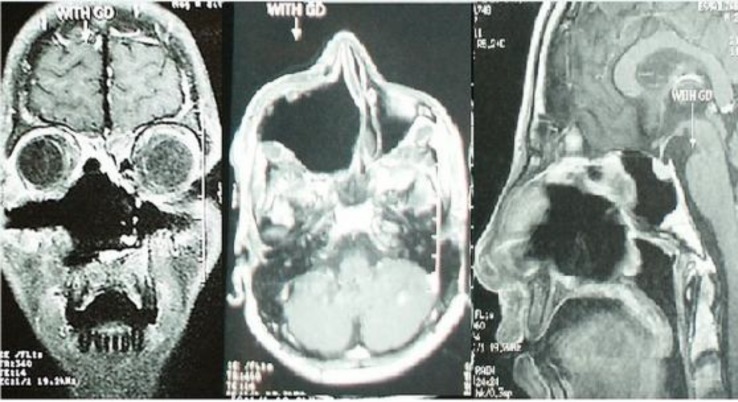
Postoperative MRI, Coronal, axial and sagittal T1 with Gd-DTPA which showed complete removal of the tumor

Postoperative MRI was done to ensure complete removal of the tumor and it showed no evidence of remnant tumor (Fig. 4).

The patient received postoperative radiotherapy and, after 1 year of follow up, no recurrence or dissemination was observed.

## Discussion

Extramedullary plasmacytoma is a rare tumor and accounts for 3-5% of all plasma cell neoplasms. Approximately 80% of them occur in the upper aerodigestive tract (7).

Extramedullary plasmacytoma chiefly affects adults older than 65-years-old and there is a male preponderance of 3:1(5).

Many cases of sinonasal plasmacytoma have been reported to date (3,5,6,8-16), few of which involved the maxillary sinus(6,12-15). Sinonasal plasmacytomas cause different symptoms depending on the sites of origins and the areas of involvement. Their usual symptoms are airway obstruction, nasal discharge, and epistaxis. They can also lead to facial pain and swelling, proptosis, visual impairment, and trismus 

Solitary extramedullary plasmacytoma shows nonspecific CT and MRI imaging features; however features like a bulky soft tissue mass or infiltrative lesion may suggest its diagnosis. The tumor does not usually become disseminated but may be locally aggressive and destructive to the adjacent structures (3).

The patient was in his thirties at the time of the diagnosis, even though this is not the typical age of occurrence. He had a two-year history of slow progressive swelling of the right cheek accompanied with right nasal obstruction and rhinorrhea. The imaging studies demonstrated a well-defined expansile soft tissue mass of the antrum with evidence of expansion and erosion of the sinus walls. The tumor showed moderate enhancement after contrast administration.

Diagnosis is based on the detection of the plasma cell tumor in an extramedullary site in the absence of bone marrow plasma cell infiltration, bone lytic lesions, and other signs of multiple myeloma. Extramedullary plasmacytoma must be distinguished from reactive plasma cell lesions and lymphoma. It should be demonstrated that the infiltration consists entirely of plasma cells and that there is no B cell component. In this regard CD138, MUMI/IRF4, CD20, and PAX5 are the most useful markers. Monoclonality and / or an aberrant plasma cell phenotype should be demonstrated with useful markers like CD19, CD56, CD27, CD117, and cyclin D1.

The diagnostic criteria of extramedullary plasmacytoma include:

1. Extramedullary tumour of clonal plasma cells

2. No M-protein in serum and/or urine

3. Normal bone marrow

4. Normal skeletal survey

5. No related organ or tissue impairment 

The following investigations should be performed in all patients diagnosed with extramedullary plasmacytoma:

1. Full blood count

2. Biochemical screen including electrolytes and corrected calcium

3. Serum immunoglobulin levels

4. Serum and urine protein electrophoresis and immunofixation

5. Serum free light chain assay

6. Full skeletal survey, including standard x-rays of the skeleton including lateral and anteroposterior cervical, thoracic and lumbar spine, skull, chest, pelvis, humeri and femora

7. MRI of spine and pelvis (or skeletal survey by MRI where this facility exists)

8. Bone marrow aspirate and trephine (4).

The endonasal biopsy of the mass, in this case, had been performed 1.5 years ago and histopathological examination had revealed a small round blue cell tumor with cells positive for the plasma cell marker CD138. Initial investigations exhibited no evidence of anemia, hypercalcemia, or renal involvement. Bence Jones protein was absent in the urine. Bone marrow examination detected no abnormality and serum protein electrophoresis showed no M component. Serum IgG, IgM, and IgA levels were normal. Skeletal survey was done and showed no focal bone disease. In addition, spinal MRI was normal.

The treatment of choice for extramedullary plasmacytoma is local radiotherapy. Although it is generally accepted that plasmacytomas are radiosensitive, there are reports of cases that do not respond to radiotherapy (9,17,18). Factors associated with poor prognosis during radiotherapy include young age, presence of bone destruction, large primary tumor (>5cm in the study of Tsang and colleges (19), recurrence of tumors, and tumors located in the sphenoid sinus, maxillary sinus, orbit, and pharynx (14).

 This patient did not respond to 28 sessions of radiotherapy; and therefore, was referred to the clinic for surgical treatment.

In comparison to previously reported cases of sinonasal plasmacytoma, a younger patient with a more advanced tumor, which did not show favorable response to radiotherapy, was observed. Nevertheless, the surgical treatment, using open approach combined with endoscopic visualization to examine the cavity following tumor removal for any remnant tumor, allowed for a complete resection. 

In 17-33% of patients with extramedullary plasmacytoma, there is a possibility of developing multiple myeloma, which requires life long follow up. The 10 year survival rate is 30-80% (5). 

 This patient received postoperative radiotherapy and no recurrence or dissemination was observed after 1 year of follow up; however, he, of course, needs longer follow-up as the progression to multiple myeloma may take several years (20).

## Conclusion

It seems that using surgical excision in persistent sinonasal EMPs after radiotherapy and especially considering early surgical intervention in cases encompassing risk factors of radiotherapy resistance before debilitating complications emerge due to tumor growth is a reasonable approach. 

This case once again reiterates the benefits of an interdisciplinary approach in the management of sinonasal extramedullary plasmacytomas.
